# CEP-1347 Boosts Chk2-Mediated p53 Activation by Ionizing Radiation to Inhibit the Growth of Malignant Brain Tumor Cells

**DOI:** 10.3390/ijms25179473

**Published:** 2024-08-30

**Authors:** Yuta Mitobe, Shuhei Suzuki, Kazuki Nakamura, Yurika Nakagawa-Saito, Senri Takenouchi, Keita Togashi, Asuka Sugai, Yukihiko Sonoda, Chifumi Kitanaka, Masashi Okada

**Affiliations:** 1 Department of Molecular Cancer Science, School of Medicine, Yamagata University, 2-2-2 Iida-Nishi, Yamagata 990-9585, Japan; 2 Department of Neurosurgery, School of Medicine, Yamagata University, 2-2-2 Iida-Nishi, Yamagata 990-9585, Japan; 3 Department of Clinical Oncology, Yamagata Prefectural Shinjo Hospital, 720-1 Kanazawa, Shinjo, Yamagata 996-8585, Japan; 4 Department of Ophthalmology and Visual Sciences, School of Medicine, Yamagata University, 2-2-2 Iida-Nishi, Yamagata 990-9585, Japan; 5 Research Institute for Promotion of Medical Sciences, Faculty of Medicine, Yamagata University, 2-2-2 Iida-Nishi, Yamagata 990-9585, Japan

**Keywords:** drug repositioning, drug repurposing, combination therapy, glioma, meningioma

## Abstract

Radiation therapy continues to be the cornerstone treatment for malignant brain tumors, the majority of which express wild-type p53. Therefore, the identification of drugs that promote the ionizing radiation (IR)-induced activation of p53 is expected to increase the efficacy of radiation therapy for these tumors. The growth inhibitory effects of CEP-1347, a known inhibitor of MDM4 expression, on malignant brain tumor cell lines expressing wild-type p53 were examined, alone or in combination with IR, by dye exclusion and/or colony formation assays. The effects of CEP-1347 on the p53 pathway, alone or in combination with IR, were examined by RT-PCR and Western blot analyses. The combination of CEP-1347 and IR activated p53 in malignant brain tumor cells and inhibited their growth more effectively than either alone. Mechanistically, CEP-1347 and IR each reduced MDM4 expression, while their combination did not result in further decreases. CEP-1347 promoted IR-induced Chk2 phosphorylation and increased p53 expression in concert with IR in a Chk2-dependent manner. The present results show, for the first time, that CEP-1347 is capable of promoting Chk2-mediated p53 activation by IR in addition to inhibiting the expression of MDM4 and, thus, CEP-1347 has potential as a radiosensitizer for malignant brain tumors expressing wild-type p53.

## 1. Introduction

Brain tumors are broadly classified into intra-axial tumors arising from the brain parenchyma and extra-axial tumors growing outside the brain parenchyma, with glioblastoma (GBM) arising from the brain parenchyma and meningioma growing outside the brain parenchyma being the most frequent and representative intracranial tumors, mainly in adults [1,2]. Among these tumors, GBM has an extremely poor prognosis, with a reported 5-year survival rate of 9.8% even when standard therapy is performed [2]. Although most meningiomas are benign, some World Health Organization (WHO) grade 2 or 3 meningiomas have high histological malignancy and penetratingly extend into the brain parenchyma. WHO grade 3 meningioma has an extremely poor prognosis and is also referred to as malignant meningioma (MM) [2,3,4].

Although surgery is the most common treatment for intracranial malignancies represented by GBM and MM, these highly invasive malignancies recur early and have a poor prognosis when treated with surgery alone [4,5,6,7]. Drug therapy is generally regarded as an essential part of cancer treatment, and although there have been numerous studies on brain tumors, there are still few effective drugs for GBM or MM [5,8,9,10]. In contrast, radiation therapy is frequently employed for the treatment of intracranial malignancies in routine practice [5,9]. However, ionizing radiation (IR) is toxic to surrounding normal cells; therefore, increasing the radiosensitivity of cancer cells is considered to be a very effective therapeutic strategy [11].

One of the mechanisms by which IR exerts its effects on cancer cells is the activation of the ataxia telangiectasia and Rad3-related (ATR)–checkpoint kinase 1 (Chk1) pathway by DNA single-strand breaks or the activation of the ataxia telangiectasia mutated (ATM)–Chk2 pathway by DNA double-strand breaks. Activated Chk1 and Chk2 phosphorylate the tumor suppressor protein p53 at different positions, resulting in the stabilization of p53 by dissociation from murine double minute 2 (MDM2), an E3 ubiquitin ligase that directs the proteasomal degradation of p53. There exists another layer of IR regulation of p53, where Chk2 phosphorylates MDM4, another negative regulator of p53 that promotes the degradation of p53 in cooperation with MDM2 and inhibits the transcriptional activity of p53 by itself. Chk2-mediated phosphorylation of MDM4 stimulates its ubiquitination and degradation by MDM2, resulting in the activation of p53. Through these mechanisms, IR activates p53 and inhibits tumor cell growth [11,12,13,14]. Although GBM and MM are both cancer types that express wild-type p53 in the majority of cases [15,16,17], p53 is negatively regulated by MDM2 and MDM4 in wild-type p53 cancer [18,19], and methods to enhance the IR sensitivity of GBM cells, with the aim of promoting the activation of p53 by the combined use of MDM2/MDM4 inhibitors, are currently being evaluated [20,21].

Conventionally, MDM2 inhibitors have primarily been evaluated as a method for the activation of p53 in wild-type p53 cancer cells; however, MDM4 inhibitors have recently been attracting increasing attention, and one of them is CEP-1347, the inhibitory effects of which on MDM4 protein expression were demonstrated in our recent study [22,23,24]. CEP-1347 is a small-molecule kinase inhibitor that penetrates the blood–brain barrier (BBB); its safety in humans is supported in the literature [25,26,27]. We recently demonstrated that CEP-1347 suppressed MDM4 expression in p53 wild-type malignant brain tumor cells, causing the activation of p53 and growth inhibition [23,24].

Given that the Chk2-mediated suppression of MDM4 expression has been identified as one of the mechanisms by which IR activates p53 [13], we hypothesized that CEP-1347 may suppress MDM4 expression jointly with IR and enhance the activation of p53 by IR. Therefore, we investigated the combined effects of IR and CEP-1347 and the underlying molecular mechanisms in GBM and MM cells. Strikingly, the results revealed that there was an unanticipated, novel mechanism of cooperation between IR and CEP-1347 to activate p53 that did not involve MDM4.

## 2. Results

### 2.1. CEP-1347 Enhances Growth Inhibitory Effects of IR in Malignant Brain Tumor Cells and Amplifies Activation of the p53 Pathway by IR

**Figure 1:**
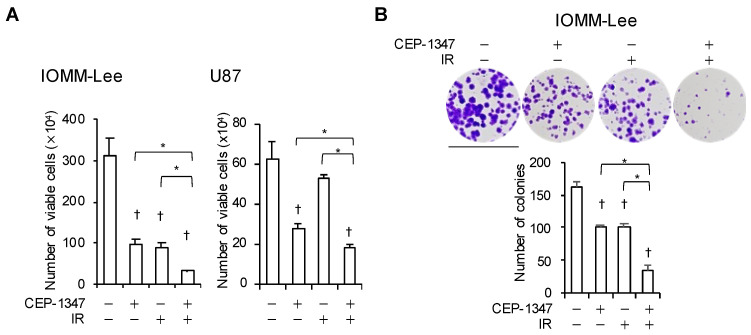
The combination of CEP-1347 and ionizing radiation (IR) inhibits the growth of malignant brain tumor cells. (**A**) IOMM-Lee and U87 cells treated without or with CEP-1347 (250 nM for IOMM-Lee and 400 nM for U87) for 1 day (IOMM-Lee) and 2 days (U87) were not irradiated or were irradiated once by X-ray (IR, 2 Gy for IOMM-Lee and 4 Gy for U87). After being cultured for 3 (IOMM-Lee) and 2 (U87) additional days, cells were subjected to the trypan blue dye exclusion assay. (**B**) IOMM-Lee cells treated without or with 250 nM CEP-1347 for 1 day were not irradiated or were irradiated once by X-ray (IR, 2 Gy). After being cultured for 1 day, cells were cultured for another 6 days in the absence of CEP-1347 for the colony formation assay. Representative images (upper panels) and the number of colonies (lower graph) are shown. Bar: 1.5 cm. Values represent the means + SDs of triplicate samples from a representative experiment. * *p* < 0.05. † *p* < 0.05 vs. cells treated without any drugs or irradiation by the Student’s *t*-test.

We previously reported that CEP-1347 inhibited the growth of GBM and MM cells, which are typical malignant brain tumor cells with the wild-type p53 gene [23,24]. In the present study, we used the MM cell line IOMM-Lee and the GBM cell line U87 with wild-type p53. The treatment with CEP-1347 inhibited the proliferation of these cells, as previously reported. In addition, a single exposure to IR of IOMM-Lee at a dose of 2 Gy and U87 at 4 Gy suppressed their proliferation. We then investigated whether the combination of the CEP-1347 treatment and IR was effective. We found that the growth of both cell types was more strongly inhibited by the CEP-1347 treatment followed by IR than by either treatment alone (Figure 1A). Furthermore, the combination suppressed clonal survival significantly more than either treatment alone (Figure 1B). These results suggest that the CEP-1347 treatment enhanced the inhibitory effects of IR on the proliferation of malignant brain tumor cells.

**Figure 2:**
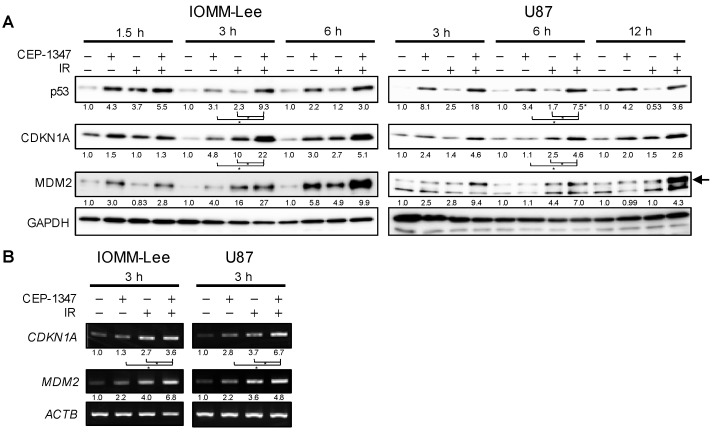
Concomitant CEP-1347 with ionizing radiation (IR) activates the p53 pathway. IOMM-Lee and U87 cells treated without or with CEP-1347 (250 nM for IOMM-Lee and 400 nM for U87) for 1 day (IOMM-Lee) and 2 days (U87) were not irradiated or were irradiated once by X-ray (IR, 2 Gy for IOMM-Lee and 4 Gy for U87). After being cultured for the indicated hours, cells were subjected to Western blot (**A**) and RT-PCR (**B**) analyses, respectively. The MDM2 specific band is indicated by an arrow. The numbers below the Western blot and RT-PCR images represent the means of the relative band intensities after each band was quantified by densitometry and normalized by the GAPDH or ACTB value, respectively. * *p* < 0.05.

CEP-1347 and IR have both been reported to increase p53 activity in tumor cells [13,23,24,28]. Therefore, we investigated whether enhancements in the growth inhibition of malignant brain tumor cells correlated with an increase in p53 activity by the use of CEP-1347 and IR combined. IOMM-Lee and U87 cells were collected after the CEP-1347 treatment followed by IR, and they were subjected to Western blotting (Figure 2A) or RT-PCR (Figure 2B). Expression levels of p53 were higher 1.5, 3, and 6 h after IR in IOMM-Lee and 3 and 6 h after IR in U87 than in cells subjected to either treatment alone (Figure 2A). Furthermore, the expression levels of CDKN1A (p21) and MDM2, the target products transcribed by p53, were also higher at the mRNA and protein levels than they were in those with either treatment alone (Figure 2A,B). These results suggest that the treatment with CEP-1347 enhanced growth inhibitory effects in association with IR-induced increases in the activation of the p53 pathway.

### 2.2. The Combination of CEP-1347 and IR Does Not Result in the Further Inhibition of MDM4 Expression

**Figure 3:**
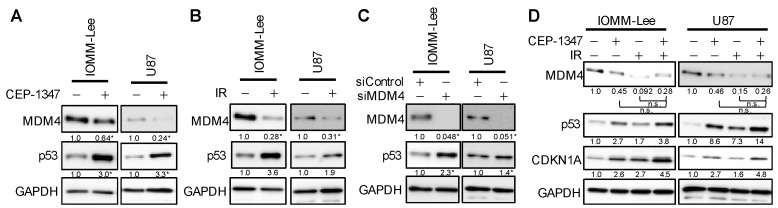
CEP-1347 and ionizing radiation (IR) each reduce MDM4 expression, whereas their combination does not result in further reductions in MDM4 expression. (**A**) IOMM-Lee and U87 cells treated without or with CEP-1347 (250 nM for IOMM-Lee and 400 nM for U87) for 1 day (IOMM-Lee) and 2 days (U87) were subjected to a Western blot analysis. (**B**) Cells were not irradiated or were irradiated once by X-ray (IR, 2 Gy for IOMM-Lee and 4 Gy for U87). After being cultured for 3 h, cells were subjected to a Western blot analysis. (**C**) Cells were transiently transfected with siControl or siMDM4. After 3 days (IOMM-Lee) and 5 days (U87), transfected cells were subjected to a Western blot analysis. (**D**) IOMM-Lee and U87 cells treated without or with CEP-1347 (250 nM for IOMM-Lee and 400 nM for U87) for 1 day (IOMM-Lee) and 2 days (U87) were not irradiated or were irradiated once by X-ray (IR, 2 Gy for IOMM-Lee and 4 Gy for U87). After being cultured for 3 h, cells were subjected to a Western blot analysis. The numbers below the Western blot images represent the means of the relative band intensities after each band was quantified by densitometry and normalized by the GAPDH value. * *p* < 0.05. n.s.: not significant.

We previously showed that CEP-1347 increased p53 expression in malignant brain tumor cells with the wild-type p53 gene by suppressing the expression of MDM4, one of the negative regulators of p53 [23,24], while IR reportedly down-regulated the expression of MDM4 by phosphorylating MDM4 through the activation of the ATM-Chk2 pathway [13]. Based on these findings, we hypothesized that the enhanced activation of the p53 pathway and growth inhibition by the combination of CEP-1347 and IR may be attributed to the increased suppression of MDM4 expression. Therefore, we investigated whether the CEP-1347 treatment or IR reduced the expression of MDM4 and increased the expression of p53 in IOMM-Lee and U87. The results obtained showed that each treatment down-regulated the expression of MDM4 and up-regulated that of p53, as previously reported (Figure 3A,B). To confirm whether the down-regulated expression of MDM4 increased the expression of p53, we examined changes in p53 after the knockdown of MDM4. Since the up-regulated expression of p53 was associated with the down-regulated expression of MDM4 (Figure 3C), CEP-1347 and IR were each expected to increase expression levels of p53 by reducing those of MDM4.

We then examined MDM4 and p53 expression levels following IR after the CEP-1347 treatment. The expression of MDM4 was decreased and that of p53 was increased by either treatment alone, as shown in Figure 3A,B. However, with the combined treatment, there was an additional increase in p53 over that with either treatment alone, as previously observed, but no further decrease in MDM4 (Figure 3D). These results suggest a molecular mechanism other than the down-regulated expression of MDM4 for the up-regulated expression of p53 by the combined treatment.

### 2.3. CEP-1347 Enhances IR-Mediated Increases in Chk2 Phosphorylation in an ATM-Independent Manner

**Figure 4:**
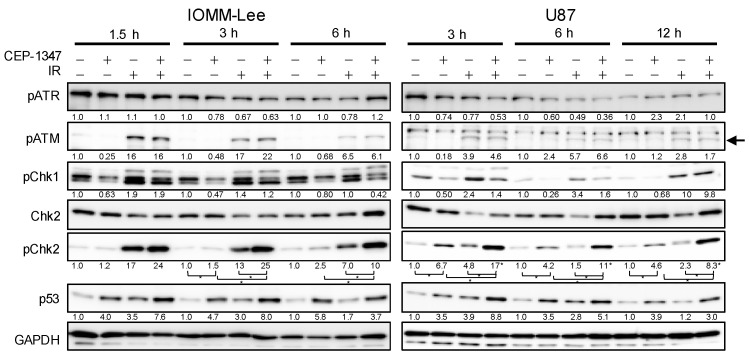
CEP-1347 promotes the phosphorylation of Chk2, but not that of ATM, induced by ionizing radiation (IR). IOMM-Lee and U87 cells treated without or with CEP-1347 (250 nM for IOMM-Lee and 400 nM for U87) for 1 day (IOMM-Lee) and 2 days (U87) were not irradiated or were irradiated once by X-ray (IR, 2 Gy for IOMM-Lee and 4 Gy for U87). After being cultured for the indicated hours, cells were subjected to a Western blot analysis. The band specific to phospho-ATM (pATM) is indicated by an arrow. The numbers below the Western blot images represent the means of the relative band intensities after each band was quantified by densitometry and normalized by the GAPDH value. * *p* < 0.05.

We examined changes in the ATM/ATR–Chk1/2 pathway, a commonly known upstream pathway of p53, to clarify why CEP-1347 and IR activated p53 independently of MDM4 expression levels. The treatment of malignant brain tumor cells by IR increased the phosphorylation of ATM and Chk2, but not that of ATR (Figure 4). On the other hand, the CEP-1347 treatment did not affect the phosphorylation of ATM/ATR or Chk1, but increased that of Chk2 (Figure 4). Furthermore, the CEP-1347 treatment enhanced the IR-mediated increase in phospho-Chk2 without affecting that in ATM phosphorylation (Figure 4). The increase in p53 expression levels generally correlated with that in phospho-Chk2 levels. These results suggest that the treatment with CEP-1347 increased p53 expression levels by enhancing Chk2 phosphorylation in coordination with IR, but in an ATM/ATR-independent manner.

### 2.4. Chk2 Activity Is Necessary for CEP-1347 Activation of p53 in Combination with IR

**Figure 5:**
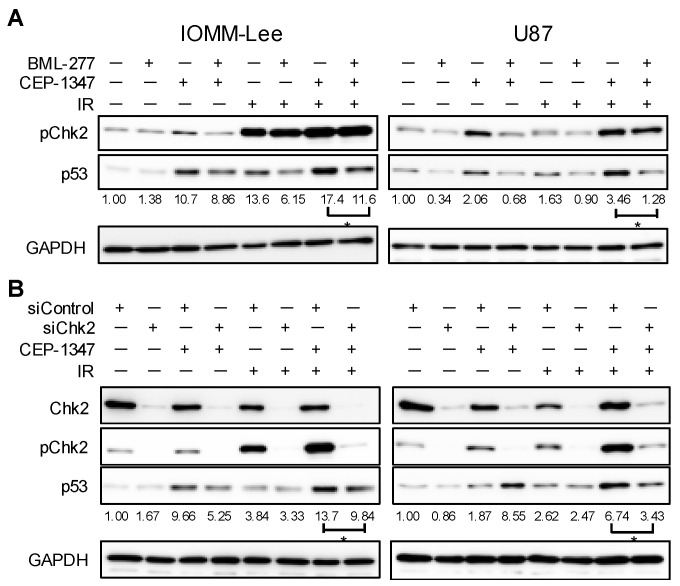
Essential role of Chk2 in the activation of p53 by combined CEP-1347 and ionizing radiation (IR). (**A**) IOMM-Lee and U87 cells treated without or with 10 μM BML-277 and CEP-1347 (250 nM for IOMM-Lee and 400 nM for U87) for 1 day (IOMM-Lee) and 2 days (U87) were not irradiated or were irradiated by X-ray (IR, 2 Gy for IOMM-Lee and 4 Gy for U87). After being cultured for 3 h (IOMM-Lee) and 6 h (U87), cells were subjected to a Western blot analysis. (**B**) IOMM-Lee and U87 cells were transiently transfected with siControl or siChk2. After 2 days, cells were treated without or with CEP-1347 (250 nM for IOMM-Lee and 400 nM for U87) for 1 day (IOMM-Lee) and 2 days (U87), and were then not irradiated or were irradiated by X-ray (IR, 2 Gy for IOMM-Lee and 4 Gy for U87). After being cultured for 3 h (IOMM-Lee) and 6 h (U87), cells were subjected to a Western blot analysis. The numbers below Western blot images represent the means of the relative band intensities after each band was quantified by densitometry and normalized by the GAPDH value. * *p* < 0.05.

We then investigated whether the increase induced in Chk2 phosphorylation by the combined treatment of CEP-1347 and IR was essential for up-regulating the expression of p53. Malignant brain tumor cells were treated simultaneously with CEP-1347 and the Chk2 inhibitor BML-277 and then by IR. Similar to previous findings, the treatment with CEP-1347 further up-regulated IR-induced p53 expression, while the presence of BML-277 attenuated the up-regulated expression of p53 (Figure 5A). We also examined p53 expression levels by performing the CEP-1347 treatment and IR under a condition in which Chk2 expression was reduced in advance by its knockdown. The knockdown of Chk2 significantly suppressed the increase induced in p53 by IR with the CEP-1347 treatment (Figure 5B). These results suggest that CEP-1347 contributed to the up-regulated expression of p53 by cooperatively acting with IR to increase Chk2 phosphorylation.

### 2.5. MDM4 Expression Is Down-Regulated by IR and CEP-1347 in Chk2-Dependent and Independent Manners, Respectively

**Figure 6:**
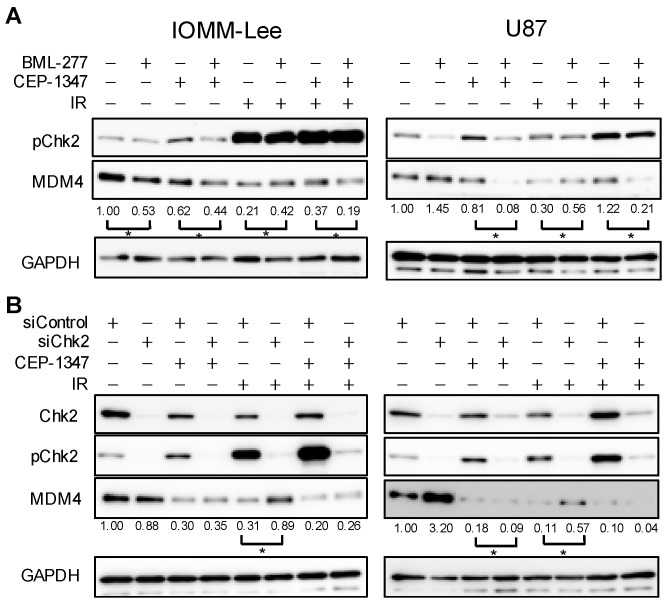
Ionizing radiation (IR) reduces MDM4 expression in a Chk2-dependent manner, whereas CEP-1347 reduces MDM4 expression in a Chk2-independent manner. (**A**) IOMM-Lee and U87 cells treated without or with 10 μM BML-277 and CEP-1347 (250 nM for IOMM-Lee and 400 nM for U87) for 1 day (IOMM-Lee) and 2 days (U87) were not irradiated or were irradiated by X-ray (IR, 2 Gy for IOMM-Lee and 4 Gy for U87). After being cultured for 3 h (IOMM-Lee) and 6 h (U87), cells were subjected to a Western blot analysis. (**B**) IOMM-Lee and U87 cells were transiently transfected with siControl or siChk2. After 2 days, transfected cells were treated without or with CEP-1347 (250 nM for IOMM-Lee and 400 nM for U87) for 1 day (IOMM-Lee) and 2 days (U87), and were then not irradiated or were irradiated by X-ray (IR, 2 Gy for IOMM-Lee and 4 Gy for U87). After being cultured for 3 h (IOMM-Lee) and 6 h (U87), cells were subjected to a Western blot analysis. The numbers below Western blot images represent the means of the relative band intensities after each band was quantified by densitometry and normalized by the GAPDH value. * *p* < 0.05.

A previous study reported that IR-induced decreases in MDM4 expression in sarcoma cells occurred in a Chk2-dependent manner [13]. Figure 3A,B show that MDM4 expression was decreased in IOMM-Lee and U87 treated with CEP-1347 or IR; however, it remains unclear whether the underlying molecular mechanisms are dependent on Chk2. Therefore, we investigated whether the reduction in MDM4 expression by the CEP-1347 treatment, IR, or their combination was dependent on Chk2 using a Chk2 inhibitor or its knockdown. In both cell types, the inhibition of Chk2 markedly attenuated the IR-induced decrease in MDM4 expression, but only slightly ameliorated, or did not affect, the reduction caused by CEP-1347 or the combined treatment (Figure 6A,B).

These results indicate that the reduction in MDM4 expression by IR was dependent on Chk2, as previously reported, whereas that induced by CEP-1347 or its combination with IR appeared to be independent of Chk2.

## 3. Discussion

CEP-1347 is a drug that we are aiming to apply to cancer therapy through drug repositioning. We previously reported that CEP-1347 exerted p53-activating and tumor growth-inhibiting effects by acting as an MDM4 inhibitor in the GBM and MM cell lines used in the present study as well as in retinoblastoma and uveal melanoma cell lines, all of which have wild-type p53 [23,24,29,30]. Furthermore, we showed that CEP-1347 activated p53 and inhibited the growth of p53 wild-type cancer cells not only when used alone, but more efficiently when used in combination with a MDM2 inhibitor [31]; however, the effects of combining CEP-1347 with IR, which is commonly employed for the treatment of GBM and MM, remain unknown. In the present study, we demonstrated that CEP-1347 and IR cooperatively induced the activation of p53 and inhibited the growth of GBM and MM cells through an unexpected mechanism as described below.

**Figure 7:**
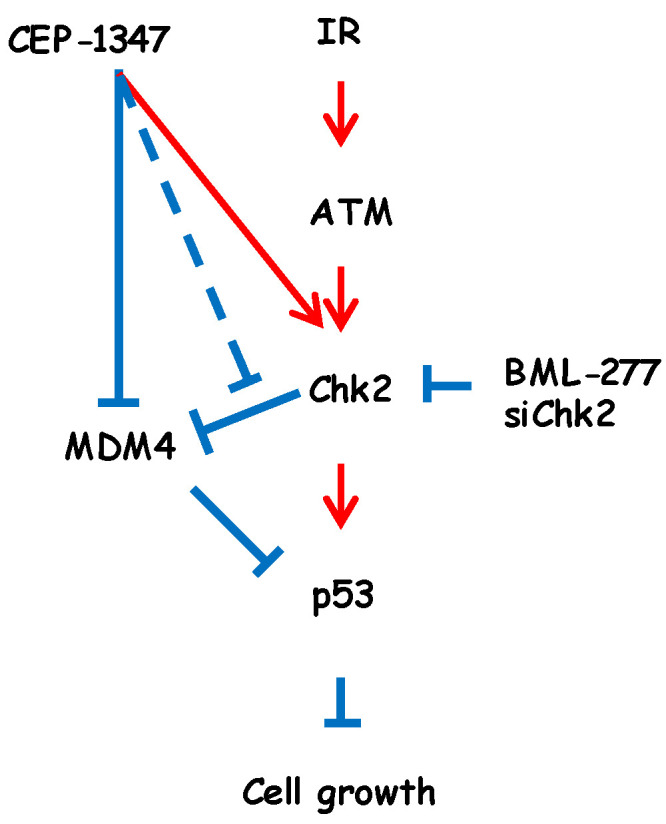
Proposed model for the molecular mechanism of CEP-1347 and IR combination therapy for malignant brain tumor cells. Red and blue lines denote activation and inhibition, respectively. Solid lines are based on current data or literature findings, while the dotted line is hypothetical.

IR, similar to CEP-1347, has been shown to reduce the expression of MDM4 in breast cancer cells and sarcoma cells, but not yet in GBM or MM cells [13]. Regarding the mechanisms responsible for this decrease by IR, the IR-induced activation of Chk2 was found to induce the phosphorylation and ubiquitination of MDM4, leading to degradation by MDM2 [13]. In the present study, we also confirmed that IR decreased the expression of MDM4 through Chk2 phosphorylation in GBM and MM, two representative intracranial malignancies. On the other hand, in the process of investigating the mechanisms underlying cooperative p53 activation by IR and CEP-1347, we found that CEP-1347 down-regulated MDM4 expression, which was consistent with our previous findings, and activated Chk2 in an ATM-independent manner (Figure 4). We initially speculated that CEP-1347, similar to IR, may exert MDM4-lowering effects through the activation of Chk2; however, the present results unexpectedly suggest that the MDM4-lowering effects of CEP-1347 were independent of Chk2. Furthermore, the decrease observed in the expression of MDM4 when CEP-1347 was used in combination with IR appeared to be independent of Chk2. Based on the present results, the molecular mechanism responsible for the up-regulated expression of p53 when CEP-1347 is combined with IR is shown in Figure 7. In brief, CEP-1347 appears to promote the up-regulation of p53 mainly by enhancing the IR-induced activation of Chk2; however, the two effects of CEP-1347, a reduction in MDM4 expression and the activation of Chk2, are independent of each other. On the other hand, since the CEP-1347-induced reduction in MDM4 expression was not enhanced by IR, CEP-1347 may suppress the Chk2-induced decrease in MDM4 expression through another mechanism (Figure 7).

The mechanisms responsible for the activation of Chk2 by CEP-1347 and the down-regulated expression of MDM4 have not yet been elucidated. Chk2 has been shown to induce the phosphorylation of p53 and activate the p53 pathway when phosphorylated at Thr68, which is located within the amino-terminal SQ/TQ cluster, by ATM activated by DNA double-strand breaks and dimerization [32,33,34]. However, as observed in the present study, other mechanisms for the activation of Chk2 that do not involve the activation of ATM, such as wild-type p53-induced phosphatase 1 (WIP1) coded by the *Protein phosphatase 1D* (*PPM1D*) gene, exist and have been reported [35,36]. WIP1 is a protein that inactivates Chk2 through its dephosphorylation [36], whereas c-Jun has been reported to function as a transcriptional enhancer that binds to the *PPM1D* promoter [37]. We previously demonstrated that CEP-1347 exerted inhibitory effects on the c-Jun N-terminal kinase (JNK) pathway in glioma stem cells [38]. Based on these findings, we speculate that CEP-1347 may activate Chk2 by down-regulating WIP1 expression through the inhibition of JNK-dependent c-Jun activity. Since the expression of MDM4 was previously shown to be positively regulated by WIP1 [37,39], further studies are warranted to investigate the possible involvement of the down-regulated expression of WIP1 in a series of p53 activation mechanisms by CEP-1347. Apart from the mechanisms of p53 activation by CEP-1347, it also remains undetermined how p53 activated by CEP-1347 and/or IR reduces the viability of malignant brain tumor cells. One likely possibility is the induction of Bax-dependent apoptosis [40]. However, since p53 has been implicated not only in apoptosis but also in a variety of non-apoptotic, non-canonical forms of cell death [41], this issue may need to be addressed extensively in future studies.

In addition to CEP-1347 having a number of molecular targets, it has already been administered to humans as a treatment for Parkinson’s disease, which provides further justification for its clinical introduction to treat neurological malignancies [26]. CEP-1347 was originally developed as a JNK pathway inhibitor to prevent neuronal death in Parkinson’s disease (PD) and was expected to become a PD treatment; however, its efficacy was not demonstrated in clinical trials. On the other hand, no clinically serious side effects were observed, assuring the safety of its use in humans [25,26,42]. In addition to its clinical safety, in vivo studies on mice showed that CEP-1347 is an agent that passes the BBB, which is an obstacle that has to be overcome in the development of therapeutic agents for brain tumors [27]. Furthermore, we examined the in vitro use of CEP-1347 in normal human fibroblasts and showed that it did not inhibit the proliferation of normal cells at least up to 500 nM, a clinically achievable concentration [23,24]. In the present study, we demonstrated that, in a clinically safe and relevant concentration range (~500 nM), CEP-1347 effectively enhanced the activation of p53 by IR and cooperated with IR to suppress tumor growth in the short term (shown using a dye exclusion assay) and in the long term (shown by a colony formation assay) (Figure 1). Therefore, CEP-1347 is considered to be a very promising treatment for p53 wild-type malignant brain tumors.

## 4. Materials and Methods

### 4.1. Reagents and Antibodies

BML-277 was purchased from Selleck (Houston, TX, USA) and was dissolved in DMSO to prepare a 10 mM stock solution. CEP-1347 was purchased from TOCRIS Bioscience (Bristol, UK) and was dissolved in DMSO to prepare a 0.5 mM stock solution. Trypan blue solution (T8154) and crystal violet (C.I. 42555) were purchased from Merck KGaA (Darmstadt, Germany). An antibody (A700-000-T) against murine double minute 4 (MDM4) was purchased from BETHYL (FORTIS LIFE SCIENCES, Waltham, MA, USA). An antibody against MDM2 (AF1244) was purchased from R&D Systems (Minneapolis, MN, USA). Antibodies against cyclin-dependent kinase inhibitor 1A (CDKN1A, p21^Waf1/Cip1^) (#2947), phospho-ataxia telangiectasia and Rad3-related (pATR) (Ser428) (#2853), phospho-checkpoint kinase 1 (pChk1) (Ser345) (#2348), phospho-Chk2 (Thr68) (#2197), Chk2 (#6334), and GAPDH (#5174) were purchased from Cell Signaling Technology, Inc. (Beverly, MA, USA). An antibody against p53 (sc-126) was purchased from Santa Cruz Biotechnology, Inc. (Santa Cruz, CA, USA). An antibody against phospho-ataxia telangiectasia mutated (ATM) (S1981) (EP1890Y) was purchased from Abcam plc. (Cambridge, UK).

### 4.2. Cell Culture

IOMM-Lee (CRL-3370), a human MM cell line, and U87 (HTB-14), a human GBM cell line, were obtained from the American Type Culture Collection (Manassas, VA, USA). U87 cells were cultured in Dulbecco’s modified Eagle’s medium (DMEM) supplemented with 10% fetal bovine serum (FBS), and IOMM-Lee cells were cultured in DMEM supplemented with 5% FBS. Culture media were supplemented with 100 U/mL penicillin and 100 μg/mL streptomycin.

### 4.3. Western Blot Analysis

A Western blot analysis was conducted as previously described [43]. Cells were harvested and washed with ice-cold phosphate-buffered saline (PBS) and lysed in RIPA buffer (10 mM Tris/HCl [pH 7.4], 0.1% sodium dodecyl sulfate [SDS], 0.1% sodium deoxycholate, 1% Nonidet P-40, 150 mM NaCl, 1 mM EDTA, 1.5 mM sodium orthovanadate, 10 mM sodium fluoride, 10 mM sodium pyrophosphate, and protease inhibitor cocktail set III [FUJIFILM Wako Chemicals, Osaka, Japan]). The lysates were immediately mixed with the same volume of 2 × Laemmli buffer (125 mM Tris/HCl [pH 6.8], 4% SDS, and 10% glycerol) and boiled at 95 °C for 10 min. After the protein concentrations of the cell lysates were measured using a BCA protein assay kit (Thermo Fisher Scientific, Waltham, MA, USA), samples containing equal amounts of protein were separated by SDS-polyacrylamide gel electrophoresis and transferred to polyvinylidene difluoride membranes. Membranes were probed with the indicated primary antibodies followed by appropriate horseradish peroxidase (HRP)-conjugated secondary antibodies as recommended by the manufacturer of each antibody. Immunoreactive bands were visualized using Immobilon Western Chemiluminescent HRP Substrate (Merck KGaA) and detected by a ChemiDoc Touch device (Bio-Rad, Hercules, CA, USA). Quantification of the bands on the membranes was performed by densitometry using ImageJ software (version 1.53k) (https://imagej.net/, accessed on 15 April 2022, National Institutes of Health, Bethesda, MD, USA).

### 4.4. Reverse Transcription–PCR Analysis

A reverse transcription (RT)–PCR analysis was conducted as previously described [24]. Total RNA was extracted from cells using Trizol (Thermo Fisher Scientific) and 1 μg of total RNA was reverse transcribed using the PrimeScript RT reagent kit (Takara Bio Inc., Shiga, Japan) according to the manufacturer’s protocol. Target genes were amplified with Quick Taq HS DyeMix (Toyobo CO., LTD., Osaka, Japan). The sequences of gene-specific primer sets are listed in Table 1.

### 4.5. Gene Silencing by siRNA

siRNAs against human MDM4 (HSS106417), human Chk2 (HSS174161), and medium GC duplex #2 of Stealth RNAi siRNA negative control duplexes were purchased from Thermo Fisher Scientific. Cells were transiently transfected with one of the siRNAs against MDM4 (siMDM4; 120–160 pmol per 6 cm dish) or Chk2 (siChk2; 120–160 pmol per 6 cm dish) or with the control siRNA (siCt; 120–160 pmol per 6 cm dish) using Lipofectamine RNAiMAX (Thermo Fisher Scientific) according to the manufacturer’s instructions.

### 4.6. Trypan Blue Dye Exclusion Assay

The numbers of viable and dead cells were measured using the trypan blue dye exclusion assay [24]. Both adherent and non-adherent cells were collected and, after centrifugation, were resuspended in PBS and stained with 0.2% trypan blue for 1 min. Viable and dead cells were identified by their ability or inability, respectively, to exclude trypan blue using a hemocytometer.

### 4.7. Colony Formation Assay

A colony formation assay was performed as previously described [24]. In brief, cells were seeded at a low colony-forming density (200 cells/12-well plate for IOMM-Lee). After being cultured as described in each figure legend, cells were fixed with paraformaldehyde (4% *v*/*v*), followed by staining with crystal violet (0.1% *w*/*v*). Colonies (consisting of ≥50 cells derived from a single cell) were counted using a microscope.

### 4.8. IR

Ionizing irradiation was conducted on cells at a dose rate of 1 Gy/min using TITAN-225S (Shimazu Systems, Shiga, Japan) in the animal facility of Yamagata University.

### 4.9. Data Reproducibility and Statistical Analysis

Western blotting, RT–PCR analyses, trypan blue dye exclusion assays, and colony formation assays were repeated at least twice with similar results, and one set of representative data is presented. Data analyses were performed using the software Microsoft Excel (Version 2402, Redmond, Washington, WA, USA). The significance of differences was assessed using the Student’s two-tailed *t*-test for comparisons of two groups; *p* values < 0.05 were considered to be significant.

## 5. Conclusions

This is the first study to demonstrate the efficacy of combining CEP-1347 and IR in activating p53 and inhibiting the growth of malignant brain tumor cells, along with the molecular mechanism therein. To realize the introduction of CEP-1347 into brain tumor therapy, an evaluation of the in vivo efficacy of the combination of CEP-1347 and IR and the establishment of a treatment schedule to maximize its therapeutic effects using animal models are awaited.

## Figures and Tables

**Table 1:** Sequences of PCR primers.

Gene Name	Forward	Reverse
*MDM2*	GGTGCTGTAACCACCTCACA	TGAGTCCGATGATTCCTGCTG
*CDKN1A*	GGGATTTCTTCTGTTCAGGCG	TGGTAGAAATCTGTCATGCTGGT
*ACTB*	CCCATGCCATCCTGCGTCTG	CGTCATACTCCTGCTTGCTG

## Data Availability

All data are contained in this article and there are no repository data.
